# Variability in market uptake of psychotropic medications in Europe reflects cultural diversity

**DOI:** 10.1186/s12913-017-2649-6

**Published:** 2017-11-06

**Authors:** J. M. Hoebert, A. K. Mantel-Teeuwisse, H. G. M. Leufkens, L. van Dijk

**Affiliations:** 1 National Institute for Public Health and the Environment (RIVM), Centre for Health Protection (GZB), P.O.Box 1, 3720 BA Bilthoven, the Netherlands; 2 Utrecht Institute for Pharmaceutical Sciences, Division of Pharmacoepidemiology and Clinical Pharmacology, Utrecht University, PO Box 80 082, 3508 TB Utrecht, The Netherlands; 3 NIVEL, Netherlands Institute for Health Services Research, P. O. Box 1568, 3500 BN Utrecht, the Netherlands

**Keywords:** Cultural diversity, Psychotropic medications, Utilization, Variation, European Union, Hofstede dimensions

## Abstract

**Background:**

In the last 20–30 years, many international studies have found substantial differences in the use of (older) psychotropic medication between European countries. The majority mentioned an important role for attitudes and beliefs towards psychotropic medication. So far, no studies have looked into the effects of cultural diversity on the use of new medications entering the market. As national cultures relate deeply to held values regarding, for example, what is seen as effective versus ineffective or safe versus dangerous, (cultural) diversity in decision making around the role of new medications in clinical practice may already be expected from the first day after market authorization.

**Methods:**

This study examined the relation between cultural diversity, described in Hofstede’s model of cultural dimensions (Power Distance, Individualism, Masculinity, Uncertainty Avoidance, Indulgence and Long-Term Orientation) and utilization of three new psychotropic medications, namely aripiprazole, duloxetine and pregabalin in Europe. Country level sales data of the case study medications were correlated to country-specific scores of Hofstede’s cultural dimensions. IMS Health’s MIDAS database has been used for sales data (converted to Defined Daily Doses/1000 inhabitants/day) for the case study medications from the market authorization date in 2004 until December 2009 for 23 EU member states.

**Results:**

Consumption of the case study medications was seen in all countries. In general, pregabalin was used more often than aripiprazole and duloxetine. In 2 years after market authorization, approximately 80% of all countries have reported use of all three molecules.

Correlations between Hofstede dimensions individualism, long-term orientation and indulgence and total use of the case study medications tended to become stronger over time, but they were only statistically significant for indulgence at two years after market authorization (rho = 0.51, *p* = 0.014) and three years after market authorization (rho = 0.54, *p* = 0.008). A more detailed analysis showed (slight) variation by molecule.

**Conclusions:**

This study is a first step in including cultural dimensions when explaining cross-national variation in the use of new medications. The results indicate that indulgence, however marginally, is a cultural aspect that relates to the utilization of new psychotropic medications, suggesting that within the cultural context, less regulation of social norms is a main factor in explaining cross-national variation in uptake of these medications.

## Background

Great differences, as well as startling similarities, can be seen when comparing global cultures. People around the globe are similar in their essential humanity: we communicate with each other, we sustain ourselves with food, and when we sleep we often dream. Yet we speak different languages, eat different foods, and dream different dreams. These are some examples of cultural diversity [1]. Culture can provide us with many answers on how and why people behave differently around the globe thereby bringing their own perspectives and values to the health care system [2, 3]. Medical care is strongly influenced by cultural norms and values ingrained over hundreds of years. Differences can show up in the types and quantities of medications doctors prescribe, the kinds and numbers of operations performed, even what blood pressure levels are thought to require treatment [4]. Cultural factors are therefore likely to play a role in explaining international variation in health care use, including utilization of medications [5]. Studies have already shown the relevance of cultural diversity in the use of antibiotics [6, 7]. These studies showed that the cross-national differences in use of antibiotics in ambulatory care are potentially explained by national cultural differences resulting from varying perceptions and influences. Psychotropic medication is another area where differences in medication use across Europe seem to be related to cultural diversity [8, 9]. In the last 20–30 years, many international studies have found substantial differences in the use of psychotropic medication among European countries [10, 11]. An important role for attitudes and beliefs towards psychotropic medication was found in those studies focusing on explanations of these differences. An international perspective on pediatric psychopharmacology (2008) stated “the cultural context seems to exert a greater influence on the identification and management of psychiatric disorders than on other areas of medication”[12].

The impact of cultural diversity might be foreseen in ‘older’ products. This is possible due to the relatively long existence and the embedment of these medications in clinical practice. However, little is known about the relevance of cultural diversity for products that recently received a market authorization. So far, no studies have looked into the effects of cultural diversity on the use of new medications entering the market. It is well known that new pharmacological therapies are challenging healthcare systems. There is an increasing need to assess their therapeutic value in relation to existing alternatives as well as their potential budget impact. [13]. Assessment of the effectiveness compared with alternative treatment(s) already plays an important role in many European member states in determining the reimbursement status of new medications [14]. However, as national cultures deeply relate to held values regarding, among others, what is seen as effective versus ineffective, safe versus dangerous, rational versus irrational and expensive versus inexpensive, diversity in decision making around the role of new medications in clinical practice may already be determined from the date of market authorization (MA). For the purpose of this study, three new psychotropic medications that were approved via the EU centralized procedure in 2004, have been selected. These medications were aripiprazole (Abilify®), duloxetine HCl (Cymbalta®/Xeristar®), and pregabalin (Lyrica®), which are (commonly) used in the treatment of schizophrenia, bipolar disorder, anxiety, and major depression [15–17]. These medications were selected because despite being clinically relevant for some patients, they are not seen as indisputable medical breakthroughs within their therapeutic areas. Nevertheless, they also can’t be seen, nor are they priced, as simply ‘me-too’ drugs. Variation in use (and effectiveness) of these medications between patients or between prescribers, and on a higher level between regions and countries, is therefore likely to be influenced by different cultural approaches in the utilization of these medications [12, 18].

The aim of the study was to assess the relation between cultural diversity and utilization of these three new medications in Europe. The utilization of these medications over time was studied (up to three years after MA) to see whether the effect of cultural diversity varied following the settlement of the new medications on the European market.

### Theoretical background

#### Cultural dimensions and expectations towards use of aripiprazole, duloxetine and pregabalin in EU member states

The effect of cultural dimensions is complex. It influences a multitude of social phenomena on the macro, such as patterned relations between large social groups, and micro level, e.g. the behavior of individualistic members of the society. The dynamic nature of culture conveys the top-down and bottom-up processes where one cultural level affects changes in other levels of culture [19]. Macro-level systems appear increasingly likely to influence the nature of micro-level interaction. Reciprocally, behavioral changes at the individual level, through bottom-up processes, become shared behavioral norms and values, modifying the culture of a macro level entity [19, 20]. Coleman’s scheme of the micro-macro linkage is one of the most useful expository vehicles for thinking about multi-level issues in social science research [20]. It indicates the relationship between macro factors (e.g. institutions) and the micro factors that underlie their causal relation (values, economic behavior). Most of the research on culture has focused on identifying the core cultural values that differentiate cultures on a macro level. An example of a popular and validated approach is Hofstede’s model of cultural dimensions [21]. He distinguished six dimensions along which cultural values can be compared with other cultures: individualism-collectivism; uncertainty avoidance; power distance (strength of social hierarchy); masculinity-femininity (task orientation versus person-orientation); long term-short term orientation and indulgence-restraint (see Table 1) [22]. As Hofstede transformed the concept of culture into quantifiable measures, it can be used in cross-national country comparative studies [6, 7].

**Table 1:** Hofstede’s definitions for the six cultural dimensions (adapted from http://geerthofstede.com/culture-geert-hofstede-gert-jan-hofstede/6d-model-of-national-culture/) and summary of hypotheses.

Power distance (PDI) relates to the extent to which the less powerful members of a society accept and expect that power is distributed unequally. It suggests that a society’s level of inequality is endorsed by the followers as much as by the leaders [7]. *Power distance is hypothesized to be positively associated with the use of the case study medications.*
Individualism (versus collectivism) (IDV) A society’s position on this dimension is reflected in whether people’s self-image is defined in terms of “I” (individualism) or “we” (collectivism). *Individualism is hypothesized to be positively associated with the use of the case study medications.*
Masculinity (versus femininity) (MAS) refers to the distribution of emotional roles between the genders. The masculinity side of this dimension represents a preference in society for achievement, heroism, assertiveness and material reward for success. Society at large is more competitive. *Masculinity is hypothesized to have no effect on the use of the case study medications.*
Uncertainty avoidance (UAI) indicates the degree to which the members of a society feel uncomfortable with uncertainty and ambiguity, and shows how comfortable society is in unstructured situations that are novel, unknown and surprising. *Uncertainty avoidance is hypothesized to have no effect on the use of the case study medications.*
Long-term (versus short-term orientation) (LTO) fosters pragmatic virtues oriented towards future rewards, in particular saving, persistence and adaptation to changing conditions. *Long-term orientation is hypothesized to be negatively associated with the use of the case study medications.*
Indulgence (versus Restraint) (IVR) Indulgence stands for a society that allows relatively free gratification of basic and natural human drives related to enjoying life and having fun. *Indulgence is hypothesized to be positively associated with the use of the case study medications.*

Based on Hofstede’s model of cultural dimensions, we assume the following relation between these cultural dimensions and the use of aripiprazole, duloxetine and pregabalin.

Power distance refers to ‘preferences of how persons with a different (social) status communicate with each other’. Albeit patient empowerment is a topic of interest in current health care, the asymmetry in status between doctors and patients remains persistent [23]. Still countries differ in their power distance and Deschepper et al. (2008) showed that power distance was positively correlated to the use of antibiotics [6]. They stated that in countries with a low power distance, a preference for deliberation about the use and perception of antibiotics between patients and doctors might affect the prescription of antibiotics [6]. Furthermore, low country scores on Power Distance may have implications for collaboration between doctors and other health care professionals, resulting in exchange of information about (optimal) medications therapy. This same phenomenon can be expected to occur with the utilization of other medications. A preference for discussion about the use and perception of new CNS medications might be favored in countries with a low power distance leading to lower use as contrasted with a ‘doctor knows best’ attitude in countries scoring high for power distance.

Individualism concerns with ‘the degree to which individuals are integrated into groups or not’. Previous literature indicates that people from individualist cultures are more likely to tolerate diversity and deviation from the norm because such cultures are extremely fragmented, with extensive individuality, due to the desirability of personal goals. Individuals are therefore less likely to be part of a group [21, 24, 25]. Diseases interfering with the CNS are more likely to cause behavior deviated from the norm. In individualistic countries CNS patients may be less stigmatized and access to appropriate services and/or use of CNS medication might be more common [24].

Masculinity can be defined as ‘the distribution of emotional roles between the genders’ and is expected to have contradictory effects on the use of these three medications. On the one hand, countries with a masculine culture may be expected to correlate negatively with the use of CNS medications, e.g. because these countries value a “live to work” ethic, rather than a “work to live” ethic, value ‘things’ more important than ‘quality of life’, are ego orientated and failing is seen as a disaster. On the other hand, these countries are strongly result oriented which hypothetically may lead to a quicker initiation of treatment in order to overcome negative effects of the disease and thus to a higher level of use.

Uncertainty avoidance has to do with ‘a society's tolerance for uncertainty and ambiguity’. In countries with high uncertainty avoidance more CNS medications may be used because doctors and societies want to avoid unstructured situations (behavior different from usual) in cases of severe CNS disorders. However, because these are new medications related to uncertainty and ambiguity with regard to effectiveness, long term side effects, and/or cost-effectiveness, doctors and patients might be less willing to use these medications compared to older, more commonly used medications in countries with a strong uncertainty avoidance. However, it should be kept in mind that uncertainties about the effectiveness of those medications may also exist.

Long-term orientation indicates the extent to which a society focuses on the future instead of the present only. Countries with a short-term orientation are normative in their thinking but also have a focus on achieving quick results whereas societies with a long term orientation show perseverance in achieving results. In addition, countries with a short term orientation may adapt to changes, such as the introduction of new medications, more rapidly than countries with a long term orientation, resulting in a higher use of these new CNS medications.

Indulgence is the feature of a society that allows satisfying, relatively free, feelings and desires.

The opposite is restraint which stands for a society that ‘suppresses gratification of needs and regulates it by means of strict social norms‘[21]. Indulgence can be expected to relate positively with the use of CNS medications, as these medications may have a rewarding effect on patients and the incentive of discontinuation might therefore be lacking. Countries with a high level of restraint might suppress the use of these new medications. Stricter regulations in these societies are either due to the higher treatment costs associated with the use of these medications and/or little additional therapeutic value or due to the risk of dependence.

Based on these hypotheses we expect that power distance, individualism, long-term orientation and indulgence may be correlated with the consumption of aripiprazole, duloxetine and pregabalin. For a summary of the hypotheses, see Table 1.

## Methods

### Data source

IMS Health’s MIDAS database was used as the source of all sales data for aripiprazole, duloxetine and pregabalin from the market authorization date in 2004 until December 2009. MIDAS is a summary of data obtained from IMS Health’s detailed audits of pharmaceutical purchases made by retailers (in 70 countries) and hospitals (in 45 countries). MIDAS contains information on sales of individual products, measured in both currency and physical units, as well as information on the product manufacturer, active ingredient, brand, form, strength, pack size, and therapeutic class.

Data used in this analysis cover sales to the retail pharmacy sector. Sales data include direct sales by suppliers to pharmacies and indirect sales via wholesalers. The retail pharmacy sector accounts for most sales in all of the countries considered in the analysis. Data were obtained for all EU member states (per 01/05/2004), except for the Mediterranean islands of Malta and Cyprus.

According to Dutch law, ethics approval was not applicable because the study involves no patient data (Medical Research (Human Subjects) Act).

Data on cultural dimensions were obtained from Hofstede [21]. Putting together national scores (from 1 for the lowest to 112 for the highest), Hofstede’s model allows international comparison between cultures using six cultural value dimensions (see Table 1) [22]. Scores are based on the analysis of a large database of employee value scores collected within various respondent groups such as employees of IBM (more than 50 countries), commercial airline pilots and students in 23 countries and civil service managers in 14 counties.

In 2010, scores on the dimensions were listed for 76 countries. The country scores on the dimensions are relative and can be assessed from: http://geerthofstede.com/research-and-vsm/dimension-data-matrix/. These dimensions have been replicated in a number of successive studies by different researchers using a variety of other matched samples of respondents [26]. Both studies that have assessed the relevance of cultural characteristics in explaining variation in the use of antibiotics, have used Hofstede’s theory of cultural dimensions [6, 7]. These dimensions furthermore provide a relatively general framework for analysis that can be easily applied because it reduces the complexities of culture and its effects on behavior into quantifiable dimensions [7]. Country scores for Hofstede dimensions as presented in ‘Cultures and Organizations 3rd edition 2010’ were available for all countries for which IMS provided utilization data [27].

### Data measurement

Volume was used as the measure of consumption and was expressed in the World Health Organization defined daily dose (DDD). The DDD is the assumed average maintenance dose per day for a medication used for its main indication in adults [28]. To allow for different population sizes, volume was expressed per 1000 inhabitants. Volume data were obtained on a quarterly level. Population statistics on number of inhabitants were obtained from the website of the United Nations, Department of Economic and Social Affairs for 2009 [29].

### Data analysis

**Fig. 1:**
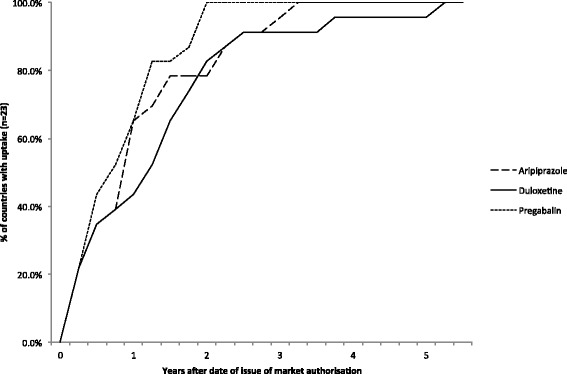
Adoption curve of the three case study medications included in the present study

For each quarter following the date of market authorization, it was assessed how many countries showed an uptake of aripiprazole, duloxetine or pregabalin. Uptake was present if the level of use was higher than 0.01 DDDs/1000inhabitants/day (arbitrary cut off point). The percentage of countries with adoption of the new medication could then be calculated and reflected in the adoption curve as shown in Fig. 1.

Spearman correlation coefficient rho was used to calculate correlations between the total usage level of aripiprazole, duloxetine and pregabalin per country at various moments in time and country scores of Hofstede dimensions. Usage level was defined as the level of utilization in DDDs/1000inhabitants/day in a certain quarter and year. A *p*-value of 0.05 or smaller was considered significant. To limit the influence of possible national system level factors, such as differences in time to reimbursement decision or national decisions about the uptake of the new medications in clinical guidelines, *t* = 0 was set country specific. In other words, t = 0 was set at the moment the first usage was seen in a country. This first usage can follow directly after MA or may be delayed due to one or more of the examples mentioned above. Stratified analyses were conducted by studying each molecule separately. As utilization of medications may vary with national wealth as expressed by gross domestic product (GDP), potential effects were examined by calculating the partial correlations controlled for GDP per capita and then compared with the unadjusted correlations.

## Results

Consumption of the case study medications was seen in all countries. One year after uptake, total utilization of aripiprazole, duloxetine and pregabalin ranged between 0.4 and 344.2 DDDs/1000inhabitants/year (Poland and Spain, respectively). This increased to range 19.0–562.1 DDDs/1000inhabitants/year (Poland and Spain, respectively), three years after uptake (see Table 2). In general, duloxetine was used more often than aripiprazole and pregabalin. Volume used in DDDs (in millions) was 1.1 (2004) to 222.1 (2009) for pregabalin, 4.2 (2004) to 256.1 (2009) for duloxetine and 1.4 (2004) tot 65.1(2009) for aripiprazole. The adoption curve (see Fig. 1) shows a similar pattern for all three molecules, i.e. 2 years after market authorization, approximately 80% of all countries reported use of all three molecules, with pregabalin having a slightly faster adoption compared to the other two medications.

**Table 2:** Total use of the case study medications and country scores on Hofstede dimensions

	Total use of the case study medications in DDDs/1000inhabitants/year				Country scores					
Country	t = 0	t = 1	t = 2	t = 3	PDI	IDV	MAS	UAI	LTO	IVR
Austria	8.9	52.0	101.6	214.5	11	55	79	70	60	63
Belgium	4.0	253.4	426.6	506.1	65	75	54	94	82	57
Czech Republic	0.5	30.7	53.6	56.5	57	58	57	74	70	29
Denmark	26.8	146.1	232.2	341.2	18	74	16	23	35	70
Estonia	10.9	33.7	52.3	45.7	40	60	30	60	82	16
Finland	2.9	123.5	269.7	452.3	33	63	26	59	38	57
France	46.0	230.9	184.8	239.9	68	71	43	86	63	48
Germany	3.8	133.7	201.9	262.3	35	67	66	65	83	40
Greece	13.9	142.4	332.3	494.9	60	35	57	112	45	50
Hungary	5.1	80.4	118.2	169.8	46	80	88	82	58	31
Ireland	8.8	152.4	254.5	350.7	28	70	68	35	24	65
Italy	6.8	127.4	203.6	262.9	50	76	70	75	61	30
Latvia	2.3	31.2	51.0	48.4	44	70	9	63	69	13
Lithuania	1.2	59.1	101.3	92.7	42	60	19	65	82	16
Luxembourg	11.0	226.1	353.2	455.2	40	60	50	70	64	56
Netherlands	5.4	91.5	138.5	176.1	38	80	14	53	67	68
Poland	0.0	0.4	10.2	19.0	68	60	64	93	38	29
Portugal	3.4	181.4	340.5	440.5	63	27	31	104	28	33
Slovak Republic	3.2	58.2	96.20	140.8	104	52	110	51	77	28
Slovenia	2.5	158.3	282.9	356.3	71	27	19	88	49	48
Spain	41.5	344.2	464.2	562.1	57	51	42	86	48	44
Sweden	0.9	76.5	202.7	336.1	31	71	5	29	53	78
United Kingdom	2.4	78.7	141.7	184.7	35	89	66	35	51	69

*PDI* power distance, *IDV* individualism, *MAS* masculinity, *UAI* uncertainty avoidance, *LTO* long-term orientation, *IVR* indulgence

t = 0: the moment first usage was seen, t = 1: one year after first usage was seen, t = 2: two years after first usage was seen and t = 3: three years after first usage was seen

Variations can be seen between the level of usage over time for the various EU member states (Table 2). Both Spain and France had a relatively high level of use one year after uptake. After three years of uptake, Spain remained to have a relatively high level of use, closely followed by Belgium, which had a relatively low consumption one year after market uptake. This same pattern (relatively low uptake after one year and a relatively high uptake after three years) can be seen for a country like Portugal, and to a lesser extent Greece.

Country scores on the Hofstede dimensions clearly varied across all countries (Table 2). Of all dimensions, masculinity was found to have the largest range (5–110) and long-term orientation the lowest range (24–83) between the studied countries. Correlations between Hofstede dimensions individualism, long-term orientation and indulgence and total use of aripiprazole, duloxetine and pregabalin tended to become stronger over time, but they were only statistically significant for indulgence at *t* = 2 (rho = 0.51, *p* = 0.014) and *t* = 3 (rho = 0.54, *p* = 0.008), see Table 3. When controlling the correlations between Hofstede dimensions and the total use of aripiprazole, duloxetine and pregabalin for wealth, Hofstede dimension indulgence remained significant (rho = 0.33, *p* = 0.007).

**Table 3:** Correlations between the total use of the case study medications and Hofstede dimensions

Cultural dimensions	Spearman correlation coefficient (Rho) (*p*-value)			
	t = 0	t = 1	t = 2	t = 3
PDI	0.122 (0.580)	.144 (0.511)	.019 (0.930)	−.024 (0.914)
IDV	0.018 (0.934)	−.022 (0.920)	−.135 (0.540)	−.170 (0.439)
MAS	0.120 (0.587)	−.078 (0.725)	−.106 (0.629)	−.086 (0.696)
UAI	.106 (0.631)	.319 (0.137)	.286 (0.186)	.265 (0.222)
LTO	−.125 (0.569)	−.267 (0.219)	−.392 (0.064)	−.407 (0.054)
IVR	.266 (0.221)	.406 (0.055)	.505 * (0.014)	.542 * (0.008)

*PDI* power distance, *IDV* individualism, *MAS* masculinity, *UAI* uncertainty avoidance, *LTO* long-term orientation, *IVR* indulgence

*= statistically significant (*p* < 0.05)

**Fig. 2:**
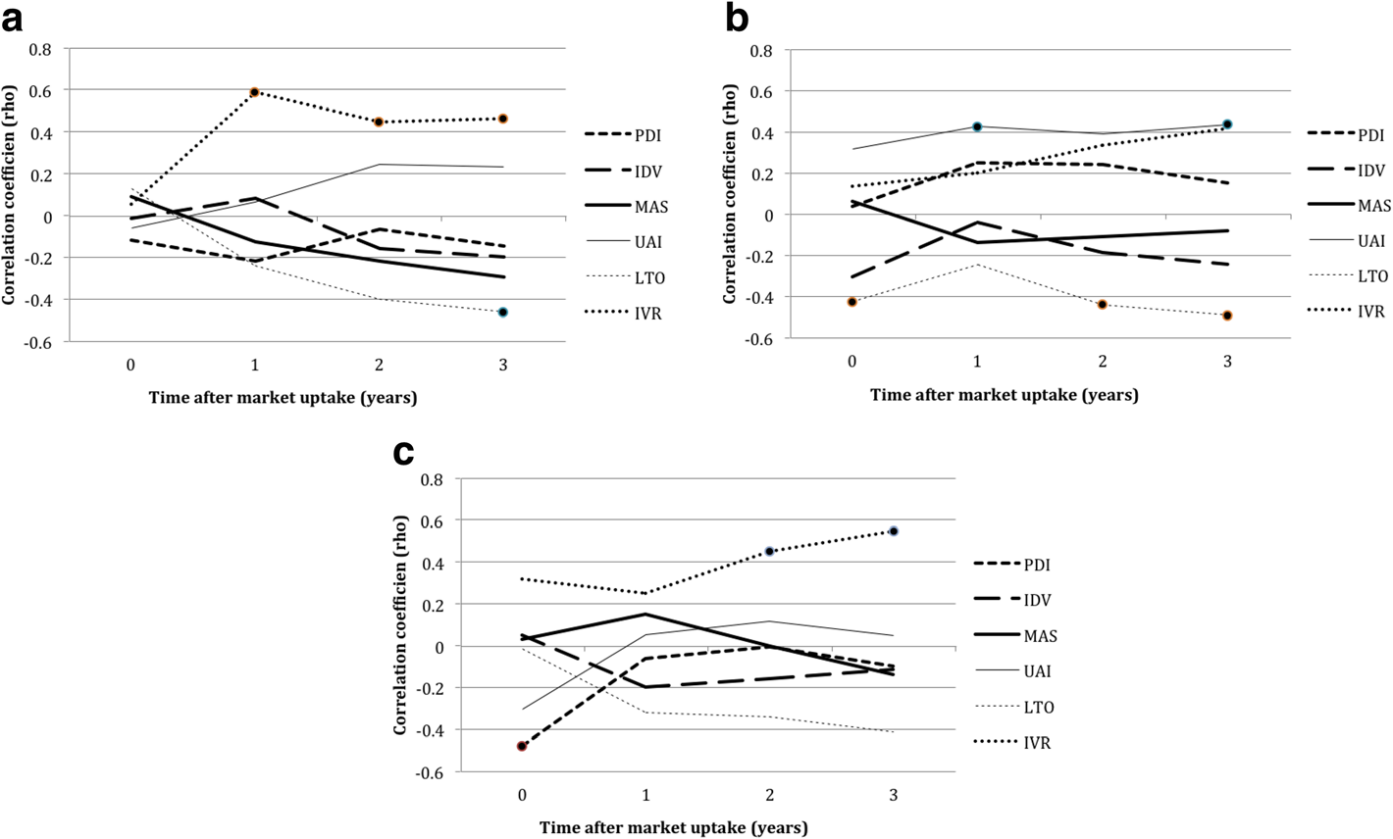
**a** Correlations between aripiprazole and Hofstede dimensions over time. Marked dots show statistically significant correlations. PDI = power distance, IDV = Individualism, MAS = masculinity, UAI = uncertainty avoidance, LTO = long-term orientation, IVR = indulgence. **b** Correlations between duloxetine and Hofstede dimensions over time. Marked dots show statistically significant correlations. PDI = power distance, IDV = Individualism, MAS = masculinity, UAI = uncertainty avoidance, LTO = long-term orientation, IVR = indulgence. **c** Correlations between pregabalin and Hofstede dimensions over time. Marked dots show statistically significant correlations. PDI = power distance, IDV = Individualism, MAS = masculinity, UAI = uncertainty avoidance, LTO = long-term orientation, IVR = indulgence

A more detailed analysis showed (slight) variation by molecule (see Fig. 2a-c). Stratified analysis revealed positive significant correlations for indulgence (at *t* = 1, 2 and 3) and a negative significant correlation for long term orientation at *t* = 3 with the use of aripiprazole. Furthermore, negative significant correlations were found for long term orientation (at *t* = 0, 2 and 3) with the use of duloxetine and a positive significant correlation at t = 1 and t = 3 for uncertainty avoidance. Positive significant correlations were also found for pregabalin and the cultural dimension indulgence at *t* = 2 and 3. A negative significant correlation was found at t = 0 for power distance and the use of pregabalin. Long term orientation was found to correlate mostly negative with the use of all case study medications (although not always significant). Indulgence was found to have the highest positive correlation for both aripiprazole and pregabalin, but not for duloxetine. Over time most of Hofstede dimensions correlated only slightly with the three case study medications. Especially in the case of pregabalin, most correlation trends over time tend off to zero.

## Discussion

This study assessed the relation between cultural diversity and utilization of three new psychotropic medications in Europe. The utilization of these medications over time was studied up to three years to explore whether the effect of cultural diversity on the total use of these new medications varied following the settlement (e.g. embedment in guidelines, reimbursement issues) of these medications on the European market.

Although homogeneity in the uptake (availability) was seen between the three molecules, large cross-national variation was seen in the level of use between one and three years after the first date of usage seen in a country. This variation came not as a surprise as many previous studies have shown consistent significant cross-national variation in older psychotropic medications utilization [8–11]. However, none of these studies linked cultural diversity to the level of use of the medications included in the studies.

The findings of a positive correlation for CNS medication use with indulgence corroborate our hypothesis with regard to this dimension and showed that (although limited) cultural diversity plays a role in the level of use of the case study medications. If we apply restraint to medical situation and decision making, policy makers and/or physicians may suppress the use of new medications, especially when treatment costs are higher and added therapeutic value is unknown compared to the already existing options and positive effects may vary between individuals. Criteria for cost-effectiveness are used for reimbursement decision in most European countries [30]. The dimension long term orientation showed a negative trend as hypothesized. Relevant elements of this dimension, such as social consumption and spending seem to exert a role in the consumption of new medications [21]. However, correlations were not found to be significant with total use of the case study medications. The trends for Hofstede dimensions ‘power distance’ and ‘masculinity’ started positive, but trended off to zero. DeSchepper et al. (2008) showed that power distance was positive correlated with the use of antibiotics suggesting that the cultural-specific way people deal with authority is an important factor in explaining cross-national differences in antibiotics use [6]. Perhaps, due to the novelty of these medications, cultural diversity in power distance has not yet shown to have an effect on the use of new medications. Finally, our hypothesis was confirmed for Hofstede dimension uncertainty avoidance (no effect on case study medications) but not confirmed for Hofstede dimension Individualism. The (slight) variation between the three case study medications furthermore shows the importance of stratified analyses. After all, when developing specific policies, it may be important to know which cultural dimension may play a role in the utilization of the medication.

This study has some limitations. The number of countries is (still) limited and the study may therefore be underpowered to detect significant differences between countries. The IMS data used in this study are sales data from the retail pharmacy sector. Any inpatient use of these medications is not included in the study. However, the bulk of the products is used in the outpatient sector. Whether the results found in this study may be due to the fact that cultural diversity does not play an important role in explaining cross-national variation in new psychotropic medication use, or that Hofstede’s model is unsuitable for new medications just entering the market remains unanswered. Hofstede’s model is based on the analysis of employees of IBM, commercial airline pilots, students and civil service managers. One could argue whether the scores based on these groups are representative and valid for prescribers and users of these medications. However, Hofstede’s model is the model most validated by research, based on rigorous cultural research [31]. It is also the most widely cited model and his observations and analyses provide researchers with highly valuable insights into the dynamics of cross-cultural diversity [31]. The model however, has not been without criticism, notably by Bhimani (1999), Harrison & McKinnon, (1999) and McSweeny (2002) [32–34]. Critiques of Hofstede’s model are mainly related to the quantitative nature of his research. For the purpose of this study, the key critique comes from Smith (2002) who argues that “…if we compare culture A and culture B on some attribute, the mean scores (macro level) that we achieve will tell us nothing about variability within each nation, nor will it tell us whether the particular individuals (micro level) whom we sampled are typical or atypical of that culture” [35].

Besides cultural values, other determinants play a role when explaining variation in medication use such as access to specialists, country’s wealth, marketing by pharmaceutical companies, differences in the types and quantities of medicines doctors prescribe, level of co-payment and date and outcome of reimbursement decisions [5]. When adjusting the correlations between Hofstede dimensions and the total use of aripiprazole, duloxetine and pregabalin for wealth, Hofstede dimension indulgence remained significant. To limit the possible effect of the moment of reimbursement decision we set *t* = 0 at the moment the first usage was seen in a country. As these medications were approved via the EU centralized procedure, no differences in market authorization dates could exist between the various countries. Unfortunately, other determinants are not (easily) measurable or available. Our limited sample size and use of a nonparametric approach precluded the possibility of a more fine-grained analysis involving other/multiple values that determine medication use.

Despite these limitations, we do believe that cultural dimensions as presented by Hofstede are a (simple but) useful tool to measure the extremely complex concept of culture. They may offer an opportunity to improve our understanding of the remarkable and unexplained cross-national variation in medication use, even for medications just entering the market. As a country’s culture is an important factor within a health care system, it should also be considered when explaining cross-national variation in the use of new medications. After all, benchmarking the level of medication use against a country with a dissimilar culture may be ineffective.

Nevertheless, more cross-national comparative research, and the availability of larger data sets including data on an individual level, is needed to better understand the relationship between cultural diversity and the use of (new) medications. As decision making never occurs in a ‘cultural vacuum’: one cannot simply transplant a policy measure from one country to another without paying attention to cultural aspects. Furthermore, the chance of (new) policies being implemented is more likely when there is sufficient base of public support.

## Conclusions

In conclusion, this study is a first step in including cultural dimensions when explaining cross-national variation in the use of new medications. The results indicate that indulgence, however marginally, is a cultural aspect that relates to the utilization of new psychotropic medications, suggesting that within the cultural context, less regulation of social norms is a main factor in explaining cross-national variation in uptake of these medications.

## Abbreviations

CNS: Central nervous system
DDD: Defined daily dose
EU: European Union
MA: Market authorization
